# Primary cilia defects in the polycystic kidneys from an ovine model of Meckel Gruber syndrome

**DOI:** 10.1186/2046-2530-1-S1-P97

**Published:** 2012-11-16

**Authors:** T Poole, C Stayner, SR McGlashan, K Parker, A Wiles, M Jennings, CG Jensen, AC Johnstone, RJ Walker, MR Eccles

**Affiliations:** 1 University of Otago, New Zealand; 2 University of Auckland, New Zealand; 3 Massey University, New Zealand

## 

Defects in primary cilia structure or function result in both common and rare ciliopathies. We have characterized an ovine model of Meckel Gruber Syndrome involving widespread dysplastic changes and cystogenesis in multiple organs. SNPchip screening identified an *MKS3* gene defect, while sequencing identified two missense mutations (I680N and I686S) in a conserved region coding for an intracellular loop of *meckelin*. We report on the nature and extent of renal primary cilia defects associated with this unique model. Columnar, cuboidal and squamous epithelia lined small cysts (1-5mm) distributed throughout the kidney, with extensive interstitial fibrosis. Immunohistochemical and SEM studies revealed a low incidence of dysmorphic epithelia cilia, including many detached near the base and shed into the cysts. Meckelin staining showed extensive vesiculation within epithelia and interstitial fibroblasts, it co-localised to the base and along the length of both attached and shed cilia, and it labeled microvesicles within cysts. In cultured fibroblasts, cilia were highly dysmorphic, ciliary incidence was similar (44% controls; 48% mutants), but ciliary length was significantly longer in mutants (17.8±1.4 cf 8.3±0.7µm; p < 0.05). Golgi markers showed extensive cytoplasmic vesiculation in mutant fibroblasts, while TEM showed Golgi distention, multivesicular bodies, intraciliary vesicles, and extracellular microvesicles. Primary cilia structure was significantly compromised in this large animal model including irregular morphology, length variation, intraciliary vesicles, cilia shedding, Golgi distention, formation of multivesicular bodies, and microvesicle release. Results suggest that disruption to the conserved isoleucines of meckelin compromises protein structure and ciliary function in this model of Meckel Gruber syndrome.

